# Unnoticed arrival of two dipteran species in Austria: the synanthropic moth fly *Clogmia albipunctata* (Williston, 1893) and the parasitic bird louse fly *Ornithoica turdi* (Olivier in Latreille, 1811)

**DOI:** 10.1007/s00436-019-06563-9

**Published:** 2019-12-13

**Authors:** Carina Zittra, Ellen R. Schoener, Rüdiger Wagner, Mike Heddergott, Georg G. Duscher, Hans-Peter Fuehrer

**Affiliations:** 1 Department of Pathobiology, Institute of Parasitology, University of Veterinary Medicine, Veterinaerplatz 1, 1210 Vienna, Austria; 2 Department of Limnology and Bio-Oceanography, University of Vienna, Althanstraße 14, 1090 Vienna, Austria; 3 Universität Kassel FB 10, Zoologie, Heinrich-Plett-Straße 40, 34132 Kassel, Germany; 4 Musée National d’Histoire Naturelle, 25 rue Münster, 2160 Luxembourg, Luxembourg

**Keywords:** Alien species, Health threat, Hippoboscidae, Psychodidae

## Abstract

In the framework of a mosquito-monitoring program conducted from 2014 to 2018, non-culicid dipteran bycatch was identified to species-level with a focus on Diptera of medical and veterinary importance as part of a biodiversity initiative and barcoding project (“Austrian Barcode of Life”). Two species hitherto not known from Austria, the regularly sampled synanthropic moth fly *Clogmia albipunctata* (Psychodidae) and a single specimen of the louse fly *Ornithoica turdi* (Hippoboscidae), were collected in Vienna and Lower Austria. We confirmed identification results using a barcoding approach and provide the first reference sequence for *O*. *turdi*.

## Introduction

Drain flies or moth flies (Diptera: Psychodidae) are small- to medium-sized non-biting midges comprising around 3000 species worldwide. *Clogmia albipunctata*, also known as the “bathroom fly” is a near-cosmopolitan and synanthropic species of tropical origin (Boumans et al. 2009). It is commonly found in bathrooms, kitchens, sewers, and sewage treatment plants and in water-filled tree holes in the tropics, Slovakia, and the USA (Oboňa and Ježek 2012a, b) where the larvae feed as scrapers of biofilm and shredders of organic material (Boumans et al. 2009). The species spread in Northern and Central Europe in the past decades and is currently widely distributed in anthropogenic habitats in tropical and temperate regions all over the world. This taxon is mostly considered a mechanical vector and indicator of poor hygiene standards, especially in hospitals, but was also found to be the cause of nasopharyngeal, intestinal and urinary myiasis in humans. The first European record was reported from Barcelona, Spain (Tonnoir 1920), under the synonym *Telmatoscopus meridionalis* (Eaton, 1894), while the distribution of this taxon was since then characterized as ranging worldwide between 40° S and 42° N (Vaillant 1971–1983). Since then, *C*. *albipuntata* was recorded in Belgium, Croatia, the Czech Republic, France, Germany, Greece, Italy incl. Sardinia, Karelian Russia, London (UK), Luxembourg, the Netherlands, Slovakia, Slovenia, Switzerland, and Spain (Ježek and Goutner 1995; Werner 1997; Ježek 2002; Withers 2005; Boumans et al. 2009; Wagner 2011; Oboňa and Ježek 2012a, b; Faulde and Spiesberger 2013; Kvifte et al. 2013; Humala and Polevoi 2015; Sivell and Irwin 2016).

The larval development of Psychodidae encompasses the egg, four larval instars, and the pupal and the adult stage and is dependent both on temperature and nutrient content (Boumans et al. 2009). *Clogmia albipunctata* females lay up to 300 eggs in moist substrate (von der Dunk 2011) and may complete its lifecycle within 17 days at temperatures ranging between 20 and 26 °C; adults live up to 10 days (Vaillant 1971). While one other group of Psychodidae, namely Phlebotominae, are vectors of the medically important protozoan *Leishmania*, *C. albipunctata* is mainly of economic importance as it can occur in large numbers in synanthropic habitats and is mainly considered nuisance pests. However, intestinal and urinary myiasis caused by *C. albipunctata* has been reported with several cases worldwide.

Urinary myiasis was documented twice from Egypt (El-Badry et al. 2014; El-Dib et al. 2017), once from the Palestinian Territories (Hjaija et al. 2018), and once from India (Sarkar et al. 2018). Intestinal myiasis was reported twice from Malaysia (Mokhtar et al. 2016; Smith and Thomas 1979), once from Japan (Tokunaga 1953), and once from Taiwan (Tu et al. 2007). Furthermore, nasopharyngeal myiasis in man was rarely reported from Africa (Mohammed and Smith 1976, Nevill et al. 1970).

Investigations of the bacterial colonization of *C. albipunctata* have demonstrated the potential to act as a mechanical vector of pathogens associated with nosocomial infections (Faulde and Spiesberger 2013). *Clogmia albipunctata* was seen several times in Austria, for example, in Lower Austria in August 2012 and years later in Upper Austria in September 2016 (cf. https://diptera.info/). Distribution and abundance data on this species in Austria are still limited. Our records and the first collected voucher specimens of this taxon contribute to the European dispersal of this species, but distribution, habitat preferences, and phenology in natural and man-made habitats in Austria as well as hospital infestations remain unknown and need to be assessed.

Hippoboscidae are robust and dorsoventrally flattened ectoparasites with a length ranging from 1.5 to 12.0 mm (Maa and Peterson, 1987). Worldwide, more than 21 genera comprising about 215 species are known, with the highest diversity in tropical and subtropical regions, while in the Nearctic region , about 13 genera containing about 31 species were recorded so far (Kock 2000, Maa and Peterson, 1987). The louse fly *O. turdi* (Latreille, 1812) is widely distributed in the Afrotropical and the Western Palearctic region (Maa 1969). This polyxenous ectoparasite had been found on a wide range of bird species among more than 57 avian genera of the orders Passeriformes, Falconiformes, Coraciiformes, Cuculiformes, and Strigiformes (cf. Maa 1969; Trilar & Krčmar, 2005). In Europe, *O. turdi* was mainly recorded on Passeriformes and once on a strigiform bird (Droz and Haenni, 2011). Although the species is known in bordering counties, e.g., in Germany, since 1990 (Kock 2000, Heddergott & Müller 2008) and in Switzerland since 2007, where it was collected on migrant Common firecrest *Regulus ignicappilus* (Droz and Haenni, 2011), it had not been found in Austria so far.

## Material and methods

The framing mosquito-monitoring project used a standardized sampling scheme across Eastern Austria based on carbon dioxide baited Biogents Sentinel (Biogents®) mosquito traps (Zittra et al. 2016). The collected samples, including bycatch, were stored at − 20 °C; bycatch was morphologically identified in the framework of the biodiversity initiative and barcoding project “Austrian Barcode of Life.” We focused on Diptera of medical and veterinary importance, and among these, we chose to work with small families first to expedite progress. Specimens of *C. albipunctata* were collected rarely as bycatch but more frequently in-house, while only a single specimen of *O. turdi* was collected using a mosquito trap. Species were identified by morphology and afterwards species identification was confirmed by analysis of the mitochondrial cytochrome oxidase subunit I gene (CO1): Genomic DNA was extracted from three legs of each specimen using the DNeasy™ Blood and Tissue Kit (Qiagen, Hilden, Germany) according to the manufacturer’s protocol. Amplification of a ~ 700-bp-long mtCO1 fragment was achieved using barcode primers LepF1 and LepR1 as well as LCO1490 and HC02198 in standard PCR protocols (Folmer et al. 1994, Hebert et al. 2004). Afterwards, purified PCR products were sequenced by a commercial company (LGC Genomics GmbH, Germany).

## Results and discussion

**Fig. 1:**
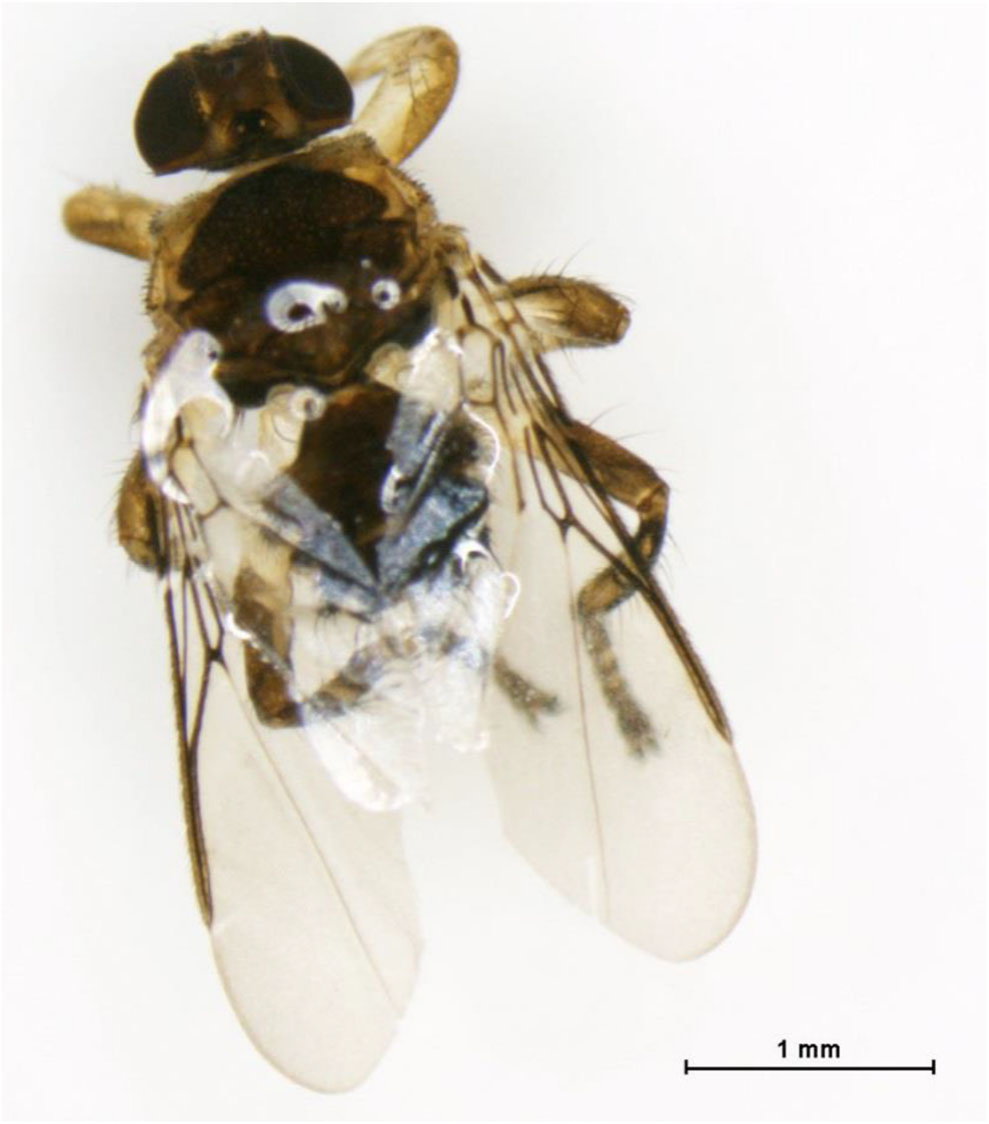
Single specimen of *Ornithoica turdi* (Diptera: Hippoboscidae), collected in Vienna in 2014

Between September 2017 and September 2018, 12 females of *C. albipunctata* were collected indoors at three locations in the district of Gänserndorf, and at a single location in the district of Korneuburg. In Vienna, a single individual of *C. albipunctata* was caught outdoors, using a carbon dioxide baited trap, in August 2017 in the 18th district of Vienna (Table 1). Whereas *C. albipunctata* was collected regularly at these sampling sites, only a single specimen o*f O. turdi* was recorded outdoors in the 14th district of Vienna in August 2014 (Fig. 1).

**Table 1:** Sampling date, storage conditions, sampling method, and locality of specimens of *Ornithoica turdi* and *Clogmia albipunctata* (*V* Vienna, *LA* Lower Austria)

ID	Taxon	Sampling date	Sampling site	Province	Longitude	Latitude	Sampling method	Storage conditions
D211	*O. turdi*	19.08.2014	11th district Vienna	V	48.176631	16.429126	Carbon dioxide trap	EtoH, − 20 °C
D171	*C. albipunctata*	02.08.2017	18th district Vienna	V	48.235873	16.335558	Carbon dioxide trap	EtoH, − 20 °C
D174	*C. albipunctata*	04.09.2017	Strasshof an der Nordbahn	LA	48.321225	16.671698	Aspirator	EtoH, − 20 °C
D173	*C. albipunctata*	01.10.2017	Korneuburg	LA	48.345079	16.334732	Manual catch	EtoH, − 20 °C
D169	*C. albipunctata*	06.11.2017	Prottes	LA	48.387095	16.736473	Aspirator	− 20 °C
D167	*C. albipunctata*	23.01.2018	Korneuburg	LA	48.345079	16.334732	Manual catch	− 20 °C
D170	*C. albipunctata*	23.01.2018	Korneuburg	LA	48.345079	16.334732	Manual catch	−20 °C
D168	*C. albipunctata*	01.05.2018	Jedenspeigen	LA	48.496363	16.875892	Manual	− 20 °C
D212	*C. albipunctata*	27.08.2018	Korneuburg	LA	48.345079	16.334732	Manual catch	EtoH, − 20 °C
D213	*C. albipunctata*	27.08.2018	Korneuburg	LA	48.345079	16.334732	Manual catch	EtoH, − 20 °C
D214	*C. albipunctata*	27.08.2018	Korneuburg	LA	48.345079	16.334732	Manual catch	EtoH, − 20 °C
D215	*C. albipunctata*	03.09.2018	Korneuburg	LA	48.345079	16.334732	Manual catch	EtoH, − 20 °C
D216	*C. albipunctata*	03.09.2018	Korneuburg	LA	48.345079	16.334732	Manual catch	EtoH, − 20 °C
D217	*C. albipunctata*	15.09.2018	Korneuburg	LA	48.345079	16.334732	Manual catch	EtoH, − 20 °C

In the case of *C. albipunctata*, molecular identification verified morphological identification of all specimens, all obtained sequences were identical (GenBank® accession number MK234696). In contrast, reference sequences were not available for *O. turdi* and we furnish the first COI sequence of this species (GenBank® accession number MK234697).

*Clogmia albipunctata* is a non-native species of tropical origin (Boumans et al. 2009), expanding its native range far to the North, transported intercontinentally by man with organic material, e.g., vegetables (Wagner et al. 2008). This taxon was reported to breed in natural tree holes in Central Europe but it is still not considered to be a biodiversity hazard at present in Austria, due to their inability to overwinter in this specialized habitats (Ježek et al. 2012; Oboňa and Ježek 2012a, b; Kvifte et al. 2013). Moreover, *C. albipunctata* is described as a year-round pest in hospitals and as a potential mechanical vector of bacterial pathogens especially of those associated with nosocomial infections, but this has not been observed in Austria so far. The regularly findings of *C. albipunctata* distributed across Lower Austria indicate a wide distribution in Austria, which should be noticed and observed vigilantly in future. However, distribution and abundance patterns of *O. turdi* in Austria are still unknown, as generally knowledge on Hippoboscidae is poor.
